# Nutritional, metabolic, and intestinal responses to dried *Opuntia ficus-indica* cladode powder in growing rabbits

**DOI:** 10.3389/fvets.2026.1991356

**Published:** 2026-10-13

**Authors:** Vincenzo Tufarelli, Caterina Losacco, Gianluca Pugliese, Francesco Giannico, Vincenzo Roselli, Maria A. Colonna, Anna Caputi Jambrenghi, Michele Schiavitto, Luca Piemontese, Letizia Passantino

**Affiliations:** 1 Department of Precision and Regenerative Medicine and Jonian Area, Section of Veterinary Science and Animal Production, University of Bari Aldo Moro, Bari, Italy; 2 Department of Human Sciences, Link Campus University, Roma, Italy; 3 Department of Pharmacy-Pharmaceutical Science, University of Bari Aldo Moro, Bari, Italy; 4 Department of Soil, Plant and Food Sciences, University of Bari Aldo Moro, Bari, Italy; 5 Italian Rabbit Breeders Association (ANCI-AIA), Foggia, Italy

**Keywords:** antioxidant status, caecal environment, digestibility, duodenal histomorphometry, *Opuntia ficus-indica*, rabbit

## Abstract

*Opuntia ficus-indica* cladodes contain fiber and bioactive compounds, but their functional value at moderate inclusion levels in rabbit diets remains unclear. This study evaluated the effects of dried *O. ficus-indica* cladode powder on nutrient utilization, redox status, lipid metabolism, caecal characteristics, and duodenal morphology in growing rabbits. Seventy male Italian White rabbits were randomly assigned at 42 days of age to a control diet (CON) or a diet containing 50 g/kg, as-fed, of dried cladode powder (OP5; *n* = 35 per treatment) and fed until 84 days of age. Dietary OP5 did not affect growth performance, feed conversion ratio, slaughter traits, or meat proximate composition (*p* > 0.05). However, crude fiber and neutral detergent fiber digestibility increased by approximately 23 and 18%, respectively (*p* < 0.001). Serum total antioxidant capacity increased by 54% (*p* = 0.001), whereas malondialdehyde, total cholesterol, and triglycerides decreased (*p* ≤ 0.006). Superoxide dismutase and hepatic enzyme activities were unaffected. Caecal pH, ammonia-N, total volatile fatty acids, and total cultivable bacterial counts did not change. Nevertheless, OP5 reduced *Escherichia coli* (*p* < 0.001) and increased *Lactobacillus* spp. (*p* = 0.029). Villus height and crypt depth increased (*p* < 0.001), while the villus height-to-crypt depth ratio decreased (*p* = 0.001), indicating duodenal remodelling. Overall, 5% dried cladode powder was well tolerated and showed potential as a functional ingredient for growing rabbits, with responses involving fiber utilization and selected antioxidant, lipid, and caecal microbial indicators without compromising productive performance.

## 1 Introduction

Nutrition plays a central role in maintaining gastrointestinal health and productive efficiency in growing rabbits. As specialized hindgut fermenters, rabbits require an adequate supply of dietary fiber to regulate digesta passage, sustain caecal microbial activity and ensure the efficient recycling of microbial nutrients through caecotrophy. These functions are particularly important during the post-weaning period, when the digestive ecosystem is still developing and animals are more susceptible to nutritional imbalances. Reductions in dietary fiber have been associated with lower caecal volatile fatty acid concentrations, increased caecal pH and ammonia levels, and a higher incidence of digestive disorders in growing rabbits (1). Therefore, the evaluation of alternative plant ingredients should consider not only their capacity to support growth, but also their effects on nutrient utilization, caecal environment and intestinal integrity.

*Opuntia ficus-indica* is a perennial cactus widely adapted to arid and semi-arid environments and may represent a locally available and climate-resilient feed resource (2). Although fresh cladodes are characterized by a high moisture content, their conversion into a dried powder facilitates preservation, standardization and incorporation into complete diets. From a nutritional perspective, cladodes generally contain low concentrations of crude protein and lipids but appreciable amounts of structural carbohydrates and minerals. Their composition, however, may vary according to cultivar, maturity, environmental conditions and processing method. In addition to their nutritional components, *Opuntia* cladodes contain several classes of bioactive compounds, including phenolic acids and flavonoid derivatives of isorhamnetin, kaempferol and quercetin (3). Their polyphenol, flavonoid and tannin fractions have been associated with measurable radical-scavenging activity (4), while studies on Mediterranean cultivars have confirmed the antioxidant potential of both young and mature cladodes (5). These characteristics suggest that dried cladode powder could be considered not only as an alternative fiber source but also as a potential functional feed ingredient.

The use of phytogenic ingredients containing antioxidant and biologically active compounds is receiving increasing attention in rabbit nutrition. Recent evidence indicated that dietary plant powders can affect systemic antioxidant status, caecal microbial populations and intestinal morphology in growing rabbits, supporting the use of an integrated approach to assess their functionality (6, 7). Nevertheless, the biological effects of phytogenic ingredients depend on their botanical origin, chemical composition, processing conditions and dietary inclusion level. Consequently, the functional properties of dried *O. ficus-indica* cladodes need to be established directly under controlled feeding conditions.

Previous studies in rabbits have mainly investigated *Opuntia* as an alternative feed resource or as a partial replacement for conventional ingredients. Zabut et al. (8) supplied fresh cactus cladodes *ad libitum* while progressively reducing the allowance of a commercial pelleted diet. The resulting decrease in growth performance at the higher replacement levels highlighted the risk of nutrient dilution when fresh cladodes are used as a major dietary component without adequate reformulation. Conversely, Pascoal et al. (9) evaluated dehydrated *O. ficus-indica* meal in nutritionally balanced diets for male growing rabbits and reported that inclusion levels of up to 30% did not adversely affect growth performance or carcass characteristics. More recently, Benali et al. (10) found that prickly pear seed cake could partially replace alfalfa at inclusion levels of 10 and 20% without impairing growth, while producing favorable changes in fiber digestibility, circulating lipid concentrations and intestinal villus measurements. However, prickly pear seed cake and cladode powder are compositionally distinct products, and their physiological effects cannot be directly extrapolated from one material to the other. Although these findings demonstrated the nutritional feasibility of using different *Opuntia* products in rabbit diets, previous investigations have predominantly focused on their use as replacement feedstuffs at relatively high inclusion levels. Evidence remains limited regarding whether a moderate inclusion of dried cladode powder in a nutritionally balanced diet can exert measurable functional effects. In particular, the combined evaluation of nutrient digestibility, systemic redox balance, lipid metabolism, caecal microbial environment and intestinal morphology may provide a more comprehensive assessment of its biological activity.

Therefore, the present study investigated the effects of incorporating dried *O. ficus-indica* cladode powder at 50 g/kg diet in male growing rabbits. Growth performance, apparent nutrient digestibility, slaughter traits and meat composition, serum antioxidant status, lipid profile and hepatic biochemical markers, caecal pH, ammonia-N, total volatile fatty acid concentration and selected microbial populations, as well as duodenal histomorphometry, were evaluated. It was hypothesized that the dietary incorporation of cladode powder would improve fiber utilization and selected indicators of antioxidant and intestinal status without adversely affecting growth performance or carcass traits.

## 2 Materials and methods

### 2.1 Animals, housing and diets

The feeding experiment was conducted at the rabbit unit of the Genetic Center of the Italian Rabbit Breeders Association (ANCI–AIA) in Volturara Appula (Foggia, Italy). Animal procedures complied with Directive 2010/63/EU and were approved by the Department of Emergency and Organ Transplantation (DiMePRe-J), University of Bari Aldo Moro (approval no. 07/2024). Seventy male Italian White (Bianca Italiana) rabbits, 42 days old and with an initial body weight of 1,037 ± 28.8 g, were randomly assigned to the control (CON) or *Opuntia*-supplemented (OP5) treatment (*n* = 35 per treatment). The feeding period lasted 42 days and ended when the rabbits reached 84 days of age. Rabbits were housed individually in galvanized-wire cages (35 × 40 × 50 cm; width × height × length) arranged in Italian-type batteries 90 cm above a concrete floor. Fresh drinking water was continuously available through automatic nipple drinkers, and feed was offered manually once daily in the morning. Husbandry and hygiene were identical for both treatments, while temperature and relative humidity were monitored throughout the study. No pharmaceutical products were administered in feed or drinking water. Animals were examined daily for clinical signs of disease and mortality. The CON group received the basal diet, whereas OP5 rabbits received a diet containing 50 g/kg, as-fed, of dried *Opuntia ficus-indica* cladode powder. The 50 g/kg inclusion level was selected as a moderate supplementation intended to assess functional responses while limiting replacement of conventional ingredients and preserving a closely matched nutrient profile between diets. The diets were formulated to provide comparable crude protein and digestible energy concentrations and to meet or exceed the nutritional requirements of growing rabbits (11), and their ingredient and chemical composition are reported in Table 1.

**Table 1:** Ingredient and chemical composition of the diets fed to rabbits.

	CON	OP5
Ingredient composition, g/kg as-fed		
Dehydrated alfalfa meal	250.0	250.0
Barley	216.2	210.2
Wheat bran	202.0	202.0
Sunflower meal, 32% CP	180.0	180.0
Dried beet pulp	110.0	60.0
Soybean oil	13.0	13.0
Soybean meal, 44% CP	10.0	16.0
Dried *Opuntia ficus-indica* cladode	-	50.0
Calcium carbonate	7.0	7.0
Sodium chloride	5.0	5.0
Vitamin–mineral premix^1^	5.0	5.0
Dicalcium phosphate	1.0	1.0
DL-methionine	0.5	0.5
L-lysine HCl	0.3	0.3
Calculated chemical composition		
Dry matter	89.48	89.25
Crude protein, % DM	17.90	17.93
Ether extract, % DM	4.19	4.22
Ash, % DM	8.77	9.15
Crude fiber, % DM	17.37	17.47
Neutral detergent fiber, % DM	38.20	37.45
Acid detergent fiber, % DM	21.25	21.05
Acid detergent lignin, % DM	5.09	5.06
Digestible energy, MJ/kg DM	11.52	11.37

CON, control diet; OP5, diet containing 50 g/kg dried *Opuntia ficus-indica* cladode powder; DM, dry matter; CP, crude protein.

1 Provided per kg of diet: vitamin A, 12,400 IU; vitamin D3, 1,550 IU; vitamin E, 32 mg; vitamin B1, 1.5 mg; vitamin B2, 5 mg; vitamin B6, 2.5 mg; vitamin B12, 0.02 mg; vitamin PP, 20 mg; vitamin K3, 2.6 mg; folic acid, 0.80 mg; pantothenic acid, 10 mg; D-biotin, 0.1 mg; choline chloride, 300 mg; MnSO₄, 152 mg; FeSO₄, 5 mg; ZnSO₄, 74 mg; CuSO₄, 5 mg; KI, 1 mg; CoSO₄, 0.2 mg; Na₂SeO₃, 0.1 mg.

### 2.2 Collection and chemical characterization of *Opuntia ficus-indica* cladodes

*Opuntia ficus-indica* cladodes were harvested in early summer in the province of Taranto, southern Italy, at the post-flowering stage. Each cladode was divided into four approximately equal pieces and dried in an oven at 30 °C for 48–72 h until constant weight. The dried cladode pieces were milled to a fine powder suitable for incorporation into the pelleted diet and stored in plastic bags at room temperature until use. The powder was analyzed in triplicate for dry matter, crude protein, ether extract, ash and crude fiber according to AOAC (12). Neutral detergent fiber, acid detergent fiber and acid detergent lignin were determined following Van Soest et al. (13). The chemical composition, total phenolic content and *in vitro* antioxidant activity of the dried powder are presented in Table 2.

**Table 2:** Chemical composition and antioxidant properties of dried *Opuntia ficus-indica* cladode powder.

Dry matter	85.33
Crude protein, % DM	5.80
Ether extract, % DM	1.56
Ash, % DM	15.44
Crude fiber, % DM	21.74
Neutral detergent fiber, % DM	32.77
Acid detergent fiber, % DM	18.97
Acid detergent lignin, % DM	1.24
TPC, mg GAE/g DW	4.57
DPPH antioxidant activity, mg GAE/g DW	0.15
ABTS antioxidant activity, mg GAE/g DW	2.21

DM, dry matter; DW, dry weight; TPC, total phenolic content; GAE, gallic acid equivalents; DPPH, 2,2-diphenyl-1-picrylhydrazyl; ABTS, 2,2′-azino-bis(3-ethylbenzothiazoline-6-sulfonic acid).

### 2.3 Extraction, total phenolic content and antioxidant activity

Phenolic compounds were extracted using a procedure adapted from Maiuolo et al. (14). Briefly, 1 g of dried cladode powder was combined with 20 mL of 80% ethanol (w/w) and agitated at 400 rpm for 24 h at room temperature. The suspension was filtered and centrifuged, after which the supernatant was recovered and stored at −20 °C pending analysis. Two independent extractions were prepared, and each was analyzed in duplicate.

Total phenolic content was quantified using a Folin–Ciocalteu assay modified from Alessandroni et al. (15) and adapted to a 96-well plate. Commercial 2 N Folin–Ciocalteu reagent was diluted with distilled water to 0.044 N. An aliquot of diluted extract (50 μL) was mixed with 100 μL of the diluted reagent and 50 μL of an 8.9% (w/v) aqueous Na₂CO₃ solution. Following incubation in the dark with shaking at 280 rpm, absorbance was measured at 765 nm using an Infinite M1000 Pro microplate reader (Tecan, Cernusco sul Naviglio, Italy). Results were calculated from a gallic acid calibration curve (8.75–280 mg/L) and expressed as milligrams of gallic acid equivalents per gram of dry weight (mg GAE/g DW).

DPPH radical-scavenging activity was measured using a modified version of the method described by Carocci et al. (16). Serial dilutions of the extract in 80% ethanol (1:2 to 1:128, v/v) were prepared, and 100 μL of each dilution was mixed with 100 μL of a freshly prepared 200 μM DPPH solution in methanol obtained from a 10 mM stock solution. After 30 min of incubation in the dark, absorbance was read at 520 nm. Activity was quantified against a gallic acid calibration curve (0.41–100 μM) and expressed as mg GAE/g DW. Two independent analytical runs were conducted in duplicate.

The ABTS radical-cation decolorization assay was adapted from Gallego et al. (17) for use in a 96-well plate. A 7 mM ABTS stock solution was prepared in phosphate-buffered saline (PBS; 50 mM, pH 7.4). A 1 mL of solution was combined with 0.5 mL of 3.5 mM potassium persulfate in PBS and kept overnight in the dark. The resulting solution was diluted with PBS to an absorbance of approximately 0.70 at 755 nm. Extract dilutions in 80% ethanol (1:50 to 1:36,450, v/v; 100 μL) were mixed with an equal volume of the ABTS working solution. After incubation for 6 min in the dark with shaking at 280 rpm, absorbance was recorded at 755 nm. Radical-scavenging activity was calculated using a gallic acid calibration curve (0.41–100 μM) and expressed as mg GAE/g DW. Two independent analytical runs were conducted in duplicate.

### 2.4 Growth performance, digestibility and productive traits

Individual body weight was recorded weekly from 6 to 12 weeks of age. Feed intake was monitored daily, and average daily gain, average daily feed intake and feed conversion ratio were calculated over the experimental period. Feed conversion ratio was derived from feed intake and body-weight gain and adjusted for mortality. Health status and mortality were recorded daily.

During the final week of the experiment, beginning at 77 days of age, 26 rabbits (*n* = 13 per treatment) were included in an apparent digestibility assessment based on the European reference procedure (18). Nylon collection nets were placed beneath the individual cages to prevent urine contamination, and feces from each rabbit were collected separately, sealed in double-layer plastic bags to limit moisture loss and immediately stored at −20 °C. For each rabbit, the material collected during the assessment was combined and homogenized. Representative subsamples were dried in a forced-air oven at 80 °C to constant weight, ground and retained for analysis. Feed and fecal samples were analyzed using the procedures described above. Apparent total-tract digestibility coefficients were estimated by the indicator method (19), with acid-insoluble ash as the internal marker (20).

At 84 days of age, 12 rabbits per treatment were randomly selected for slaughter measurements. Animals selected in the afternoon were transported in small groups to a nearby commercial slaughterhouse the following morning. Slaughter and carcass measurements were completed within 2 h of arrival in accordance with World Rabbit Science Association recommendations (21). Rabbits were weighed immediately before slaughter, electrically stunned and exsanguinated. The skin, gastrointestinal tract and distal portions of the limbs were removed. Hot carcasses, which retained the head, thoracic organs, liver, kidneys and perirenal and scapular fat, were weighed and then chilled for 24 h at 4 °C in a ventilated cold room. Carcasses were reweighed after chilling, and dressing percentage was calculated as hot carcass weight divided by preslaughter body weight × 100. Samples of the *Longissimus lumborum* muscle were analyzed in triplicate for moisture, protein, lipids and ash using AOAC (12) procedures.

### 2.5 Serum lipid profile, hepatic enzyme activities and antioxidant status

At 84 days of age, blood was collected from the marginal ear vein of 12 rabbits per treatment approximately 1 h before the usual feeding time. Samples were transferred to sterile tubes and centrifuged; the recovered serum was stored frozen at −20 °C until analysis. Serum total cholesterol and triglyceride concentrations and aspartate aminotransferase (AST) and alanine aminotransferase (ALT) activities were determined. Total antioxidant capacity (TAC), malondialdehyde (MDA) concentration and superoxide dismutase (SOD) activity were also measured using commercial assay kits (Randox Laboratories Ltd., Crumlin, UK) according to the manufacturer’s instructions. Each serum sample was analyzed in triplicate.

### 2.6 Caecal fermentation and bacterial populations

At slaughter, caecal contents were gently expressed, thoroughly mixed and divided into aliquots. A weighed portion was diluted 1:10 (w/v) in ice-cold PBS (pH 7.0), homogenized with a portable glass homogenizer and centrifuged at 5000 × g for 20 min at 4 °C. The supernatant was recovered and maintained at 4 °C pending analysis. The pH of fresh caecal digesta was measured with a portable pH meter. Volatile fatty acids were quantified by gas chromatography as described by Abu Hafsa et al. (22), whereas ammonia-N was measured with an automated distillation unit.

Separate aliquots of caecal content were transferred to sterile plastic tubes and stored at −80 °C for microbiological analysis. Cultivable populations were enumerated on Plate Count Agar for total bacterial counts, chromogenic coliform agar for *Escherichia coli* and de Man–Rogosa–Sharpe agar for *Lactobacillus* spp., following Maturin and Peeler (23). Colonies were counted with an optical colony counter, and results were expressed as colony-forming units per gram of caecal content.

### 2.7 Duodenal histomorphometry

Duodenal tissue was collected at 84 days of age from all rabbits selected for slaughter. A segment approximately 2 cm long was excised 3 cm distal to the pylorus, gently flushed with 0.9% saline and fixed in freshly prepared 10% neutral-buffered formalin. Samples were dehydrated through graded alcohols, cleared and embedded in paraffin. Sections 5–7 μm thick were mounted on glass slides and stained with hematoxylin and eosin (Merck, Darmstadt, Germany).

For each rabbit, 25 well-oriented villi with an intact *lamina propria* were randomly selected for morphometric measurements. The recorded variables were villus height, villus width and crypt depth. Villus height was measured from the villus tip to the villus–crypt junction, and crypt depth from this junction to the base of the crypt; the villus height-to-crypt depth ratio was subsequently calculated (24). Measurements were obtained at 10 × and 25 × magnification using an X-Series image-analysis system (Alexasoft).

### 2.8 Statistical analysis

Data were analyzed by one-way ANOVA using the GLM procedure of SAS [version 9.2; (25)], with dietary treatment included as the fixed effect. The individual rabbit was the experimental unit for body weight, digestibility, slaughter, serum, caecal and histomorphometric outcomes; because rabbits were housed individually, each cage–rabbit unit was used for feed intake and feed conversion ratio. Microbial counts were log₁₀-transformed before analysis, and distributional assumptions were assessed by inspection of normal probability plots. When applicable, least-squares means were compared using Tukey’s honestly significant difference test. Results are reported as least-squares means with the pooled standard error of the mean. Statistical significance was declared at *p* ≤ 0.05.

## 3 Results

### 3.1 Growth performance, nutrient digestibility, productive traits

Growth performance and apparent nutrient digestibility coefficients are presented in Table 3. Initial body weight was comparable between the CON and OP5 groups (*p* > 0.05). Dietary treatment did not affect final body weight, average daily gain, average daily feed intake, or feed conversion ratio in CON and OP5 rabbits (*p* > 0.05). The apparent digestibility coefficients of dry matter, organic matter, crude protein, and ether extract did not differ between treatments (*p* > 0.05). Conversely, the apparent digestibility coefficient of crude fiber was higher in OP5 than in CON rabbits (*p* < 0.001), and a similar response was observed for neutral detergent fiber digestibility, which was higher in the OP5 group (*p* < 0.001). Slaughter traits and meat composition are reported in Table 4. Slaughter weight, hot carcass weight, and dressing percentage did not differ between the CON and OP5 groups (*p* > 0.05). Similarly, dietary treatment had no effect on meat moisture, protein, lipid, or ash (*p* > 0.05).

**Table 3:** Growth performance and apparent digestibility coefficients of rabbits fed diets containing dried *Opuntia ficus-indica* cladode powder.

Item	CON	OP5	SEM	*p*-value
No. of rabbits	35	35		
Initial body weight^1^, g	1,031	1,040	19.7	>0.05
Final body weight^1^, g	2,107	2,154	44.9	>0.05
Average daily gain^1^, g/day	25.62	26.52	0.88	>0.05
Average daily feed intake^2^, g/day	110	113	0.91	>0.05
Feed conversion ratio^2^, g/g	4.29	4.26	0.19	>0.05
Apparent digestibility				
No. of rabbits	13	13		
Dry matter	0.602	0.619	0.021	>0.05
Organic matter	0.619	0.625	0.013	>0.05
Crude protein	0.746	0.753	0.026	>0.05
Ether extract	0.822	0.830	0.045	>0.05
Crude fiber	0.224	0.276	0.005	<0.001
Neutral detergent fiber	0.331	0.389	0.009	<0.001

CON, control diet; OP5, diet containing 50 g/kg dried Opuntia ficus-indica cladode powder; SEM, pooled standard error of the mean. ^1^Individual-rabbit data. ^2^Individual-cage data.

**Table 4:** Slaughter traits and meat composition of rabbits fed diets containing dried *Opuntia ficus-indica* cladode powder.

Item	CON	OP5	SEM	*p*-value
Slaughter weight (SW), g	2048	2069	51.1	>0.05
Hot carcass weight, g	1,335	1,350	40.2	>0.05
Dressing percentage, %	65.19	65.25	0.89	>0.05
Meat nutritional composition, %				
Moisture	72.8	73.0	0.59	>0.05
Protein	23.9	23.5	0.47	>0.05
Lipids	1.75	1.72	0.28	>0.05
Ash	1.55	1.78	0.21	>0.05

CON, control diet; OP5, diet containing 50 g/kg dried *Opuntia ficus-indica* cladode powder; SEM, pooled standard error of the mean.

### 3.2 Serum variables, caecal characteristics and duodenal histomorphometry

Serum antioxidant status, lipid profile, and hepatic biochemical markers are shown in Table 5. Total antioxidant capacity was higher in OP5 than in CON rabbits (*p* = 0.001), whereas malondialdehyde concentration was lower in the OP5 group (*p* = 0.006). Serum superoxide dismutase activity was unaffected by treatment (*p* > 0.05). Rabbits receiving the OP5 diet had lower serum total cholesterol (*p* = 0.001) and triglyceride concentrations (*p* = 0.002) than CON rabbits. No treatment-related differences were detected in serum AST or ALT activity (*p* > 0.05).

**Table 5:** Serum antioxidant status, lipid profile and hepatic biochemical markers of rabbits fed diets containing *Opuntia ficus-indica* cladode powder.

Item	CON	OP5	SEM	*p*-value
TAC, ng/mL	1.33	2.05	0.080	0.001
MDA, nmol/mL	1.56	1.11	0.072	0.006
SOD, U/mL	0.17	0.15	0.010	>0.05
Total cholesterol, mg/dL	101.2	72.6	3.15	0.001
Triglycerides, mg/dL	118.8	101.2	2.03	0.002
AST, U/L	60.4	57.6	12.55	>0.05
ALT, U/L	39.4	41.7	2.75	>0.05

CON, control diet; OP5, diet containing 50 g/kg dried *Opuntia ficus-indica* cladode powder; TAC, total antioxidant capacity; MDA, malondialdehyde; SOD, superoxide dismutase; AST, aspartate aminotransferase; ALT, alanine aminotransferase; SEM, pooled standard error of the mean.

Caecal characteristics and microbial populations are presented in Table 6. Caecal pH, ammonia-N concentration, and total VFA concentration did not differ between the CON and OP5 groups (*p* > 0.05). Total microbial counts were also unaffected by dietary treatment (*p* > 0.05). The caecal count of *E. coli* was lower in OP5 than in CON rabbits (*p* < 0.001). Conversely, the count of *Lactobacillus* spp. was higher in the OP5 group (*p* = 0.029).

**Table 6:** Caecal characteristics and bacterial populations of rabbits fed diets containing dried *Opuntia ficus-indica* cladode powder.

Item	CON	OP5	SEM	*p*-value
Caecal characteristics				
pH	6.00	6.05	0.07	>0.05
Ammonia-N, mmol/L	4.22	4.31	0.59	>0.05
Total VFA, mmol/L	72.4	73.2	3.22	>0.05
Caecal microbial populations				
Total count, ×10^6^ CFU/g	16.9	17.4	1.55	>0.05
*Escherichia coli*, log CFU/g	3.94	3.01	0.11	<0.001
*Lactobacillus* spp., log CFU/g	2.41	3.12	0.22	0.029

CON, control diet; OP5, diet containing 50 g/kg dried *Opuntia ficus-indica* cladode powder; VFA, volatile fatty acids; CFU, colony-forming units; SEM, pooled standard error of the mean.

Duodenal histomorphometric measurements are reported in Table 7. Villus height was greater in OP5 than in CON rabbits (*p* < 0.001), whereas villus width did not differ between groups (*p* > 0.05). Crypt depth was greater in OP5 rabbits (*p* < 0.001), while the villus height-to-crypt depth ratio was lower than that observed in CON rabbits (*p* = 0.001).

**Table 7:** Duodenal histomorphometry of rabbits fed diets containing dried *Opuntia ficus-indica* cladode powder.

Item	CON	OP5	SEM	*p*-value
Villus height, μm	702	744	8.02	<0.001
Villus width, μm	70.1	72.0	2.43	>0.05
Crypt depth, μm	120.5	133.1	2.03	<0.001
Villus height:crypt depth ratio	5.83	5.59	0.045	0.001

CON, control diet; OP5, diet containing 50 g/kg dried *Opuntia ficus-indica* cladode powder; SEM, pooled standard error of the mean. Each value represents the mean of 12 rabbits per treatment.

## 4 Discussion

At the inclusion level of 50 g/kg diet, dried *Opuntia ficus-indica* cladode powder did not affect growth performance, slaughter traits, or meat composition, but selectively improved fiber digestibility and modulated serum antioxidant and lipid indicators, caecal bacterial populations, and duodenal morphometry. The response therefore supported its potential as a functional dietary ingredient rather than as a growth-promoting additive.

### 4.1 Growth performance, nutrient digestibility, and slaughter traits

The lack of treatment effects on final body weight, average daily gain, feed intake, and feed conversion ratio indicates that the 5% inclusion level was well accepted and did not affect the nutritional value of the complete diet to an extent that limited growth. This agrees with Pascoal et al. (9), who found no impairment of growth performance when dehydrated *O. ficus-indica* meal was included at substantially higher levels in balanced diets for growing rabbits. The finding is also consistent with the study of Benali et al. (10), in which prickly pear seed cake partially replaced alfalfa without altering rabbit body weight or weight gain. Although seed cake and cladode powder are compositionally different, the concordant responses suggest that properly formulated diets can accommodate several *Opuntia*-derived ingredients without adversely affecting productive performance. Evidence from another livestock species is directionally similar: Moula et al. (26) reported that 5 or 10% *O. ficus-indica* powder did not adversely affect growth or carcass traits in broiler chickens. The comparison with poultry should nevertheless be regarded as supportive rather than directly transferable because digestive physiology differs markedly between birds and rabbits.

The 50 g/kg level was deliberately lower than the 10 and 20% seed-cake inclusions tested by Benali et al. (10) and the inclusion rates of up to 30% dehydrated cladode meal evaluated by Pascoal et al. (9); it also differed from the fresh-cladode replacement strategy of Zabut et al. (8), in which the commercial pelleted feed allowance was progressively reduced. These comparisons support the use of 50 g/kg as a moderate functional inclusion that minimizes nutrient dilution. However, the single-level design cannot establish whether the digestibility, redox, lipid, microbial, or morphometric responses are linear, threshold-dependent, or maximal at 50 g/kg. Dose–response trials are needed to identify the minimum effective and optimal inclusion levels.

The clearest nutritional response was the selective increase in fiber digestibility. Relative to control-diet, feeding *O. ficus-indica* powder increased the apparent digestibility coefficient of crude fiber by approximately 23% and that of NDF by approximately 18%, whereas the digestibility of dry matter, organic matter, crude protein, and ether extract remained unchanged. Because the calculated crude fiber concentrations of the diets were almost identical and the calculated NDF concentration was slightly lower in OP5, the response cannot be explained simply by a greater total fiber supply. Instead, it is compatible with a difference in fiber source, accessibility, or fermentability. Dehydrated cladode meal contains structural carbohydrates together with non-fibrous polysaccharides and a low lignin fraction, and Pascoal et al. (9) reported appreciable fiber utilization for this ingredient in rabbits. Similarly, Benali et al. (10) observed improved fiber digestibility at the lower inclusion level of prickly pear seed cake. These comparisons support, but do not prove, the possibility that the physical and chemical characteristics of the *Opuntia* fraction facilitated microbial access to dietary fiber. The composition of the powder provides further context for this response. It contained 32.7% NDF and 18.9% ADF on a dry-matter basis, but only 1.24% acid detergent lignin (Table 2), indicating a low lignified fraction relative to total cell-wall material. *Opuntia ficus-indica* cladodes also contain pectic mucilage and other water-soluble polysaccharides that are not resolved by crude-fiber or NDF measurements (27, 28). In rabbit caecal inoculum, pectin is fermented more rapidly and extensively than cellulose (29), and dietary pectin has been associated with higher crude-fiber and NDF digestibility in growing rabbits (30). Thus, a readily fermentable pectic/mucilage fraction, together with low lignification, may have increased microbial access to and disappearance of fiber. This interpretation remains provisional because pectin, soluble fiber, mucilage, particle-size distribution, and fermentation kinetics were not measured in the present study. Moreover, the cladode powder contained 85.33% dry matter, corresponding to 14.67% residual moisture. Although the material was oven-dried to constant weight before milling, water activity, fungal counts, and mycotoxin concentrations were not determined; therefore, its microbiological stability during extended storage cannot be inferred from the present data. Future studies should define storage duration and packaging and monitor water activity, fungal growth, and relevant mycotoxins, particularly when residual moisture exceeds about 12%.

The absence of differences in slaughter weight, hot carcass weight, and dressing percentage is coherent with the similar growth rate and feed conversion of the two rabbit groups. Likewise, unchanged meat moisture, protein, lipid, and ash concentrations indicate that the functional responses observed in serum and the digestive tract were not accompanied by a detectable alteration in basic meat composition. Pascoal et al. (9) also reported that dehydrated forage cactus meal did not compromise carcass characteristics in growing rabbits, while Benali et al. (10) found broadly preserved productive traits with prickly pear seed cake. Thus, the current results strengthen the evidence that *Opuntia*-derived feed materials can be incorporated into nutritionally balanced rabbit diets without a penalty in slaughter output.

### 4.2 Antioxidant, lipid, and caecal responses

The increase in serum total antioxidant capacity (~54%) together with the reduction in malondialdehyde (29%) provides the strongest evidence of a systemic antioxidant effect. Total antioxidant capacity reflects the combined activity of several circulating antioxidant components, whereas malondialdehyde is commonly used as an indicator of lipid peroxidation. Their simultaneous change therefore suggests greater antioxidant protection and lower oxidative damage in OP5 rabbits. However, superoxide dismutase activity was unchanged, showing that the response did not involve a generalized increase in all measured antioxidant defenses. This effect is biologically plausible in light of the total phenolic content and *in vitro* radical-scavenging activity measured in the cladode powder used in the present study. Cladodes of *Opuntia* spp. contain phenolic acids and flavonoid derivatives, although their qualitative and quantitative profiles vary with genotype, maturity, environment, and processing (3–5). The current trial did not identify individual phenolics or measure their absorption and metabolism, so these compounds should be considered candidate contributors rather than confirmed mediators. A comparable increase in antioxidant capacity and reduction in lipid peroxidation were reported in rabbits receiving another phenolic-rich plant powder (7), supporting the general feasibility of modulating redox status with phytogenic feed ingredients while not establishing an *Opuntia*-specific mechanism.

Phenolic acids and flavonoids may contribute through direct radical-scavenging and metal-chelating reactions and through indirect effects on redox-sensitive cellular pathways. Italian *O. ficus-indica* cladodes have been shown to contain diverse phenolic compounds together with *in vitro* antioxidant activity (31), while isolated mucilage and pectic fractions from cladodes also display radical-scavenging and reducing activities (27). Nevertheless, the present study did not quantify individual compounds in feed or serum, their bioavailability, non-enzymatic antioxidants, or the expression or activity of additional antioxidant enzymes. The unchanged SOD activity therefore cautions against attributing the TAC and MDA responses to a specific compound or pathway.

The OP5 also reduced serum total cholesterol by approximately 28% and triglycerides by approximately 15%. Similar directional responses were reported by Benali et al. (10), particularly at the higher inclusion of prickly pear seed cake in rabbits, and by Moula et al. (26) in broilers fed *O. ficus-indica* powder. Potential explanations include effects of the soluble polysaccharide fraction on intestinal viscosity, bile acid recycling, microbial metabolism, or hepatic lipid handling, as well as actions of phenolic compounds. None of these mechanisms was directly measured here. Moreover, the unchanged apparent digestibility of ether extract argues against a major reduction in total dietary lipid absorption under the conditions of this experiment. The hypolipidemic response should therefore be regarded as a robust phenotypic observation whose mechanism remains to be established. For lipid metabolism, soluble pectic polysaccharides could increase digesta viscosity and modify the enterohepatic recycling of bile acids, whereas fermentation products and phenolic compounds could influence hepatic cholesterol and triglyceride metabolism. Prickly-pear pectin modified LDL metabolism in cholesterol-fed guinea pigs (32), and lyophilized *O. ficus-indica* cladodes reduced plasma cholesterol, low-density lipoprotein, and triglycerides in hypercholesterolemic rats (33). These studies provided biological plausibility but cannot establish the mechanism in healthy growing rabbits because species, metabolic status, form of the *Opuntia* preparation, and dose differed. In addition, the present trial did not measure digesta viscosity, bile-acid or fecal-sterol excretion, individual VFA profiles, hepatic lipid-related gene expression, or the pectin content of the tested powder.

Serum AST and ALT activities were unaffected. In the context of the present feeding period, this finding provides no biochemical indication of hepatocellular injury associated with OP5. It does not, however, demonstrate hepatoprotection, because liver histology and additional markers of hepatic function were not evaluated.

The unchanged caecal pH, ammonia-N, total VFA concentration, and total cultivable microbial count indicate that OP5 did not disrupt the overall caecal fermentation environment. Against this stable background, the 0.93-log reduction in *E. coli* and the 0.71-log increase in *Lactobacillus* spp. suggest a selective shift in the cultivable populations examined rather than a non-specific increase or suppression of microbial biomass. A similar direction of change in these two bacterial groups was observed in growing rabbits supplemented with horehound powder (7), despite differences in botanical origin and inclusion level.

The absence of a corresponding change in total volatile fatty acid concentration does not contradict the digestibility response. Caecal VFA concentration is a net value determined by production, absorption, and digesta passage and was measured at a single sampling time. Consequently, a higher disappearance of fiber may occur without a detectable increase in the standing concentration of total VFAs. Individual VFA production rates and molar proportions would be needed to determine whether OP5 altered specific fermentation pathways. This distinction is particularly important in rabbits, in which fiber type and fermentability influence both caecal activity and digestive health (1).

Several non-exclusive mechanisms may have contributed. More accessible fermentable carbohydrate could favor bacteria able to use these substrates, while phenolic and other secondary metabolites could exert selective antimicrobial effects. Extracts from mature cactus-pear cladodes have shown antioxidant and antibacterial activity against selected bacterial pathogens *in vitro* (34), providing mechanistic plausibility. Nevertheless, an extract-based *in vitro* effect cannot be assumed to occur after feeding intact cladode powder, and the present culture data do not demonstrate direct inhibition of *E. coli* by a specific compound.

The microbial findings should therefore be described as a potentially favorable modulation of selected caecal populations rather than as proof of a globally improved microbiome or eubiosis. Only two bacterial groups and a total count were quantified by culture-based methods, and no microbial community profiling, functional gene analysis, or pathogen challenge was performed. In addition, total VFA concentration alone does not reveal whether acetate, propionate, butyrate, or minor acids changed as molar proportions. Broader microbiome analysis combined with individual VFA profiles would be required to connect the observed fiber digestibility response with specific microbial functions.

### 4.3 Duodenal histomorphometry

The duodenal mucosa showed a mixed morphometric response. Villus height increased by approximately 6%, a change compatible with a larger nominal absorptive surface. Crypt depth increased more markedly, by approximately 10%, and consequently the villus height-to-crypt depth ratio decreased by approximately 4%. Greater villus dimensions have also been reported with prickly pear seed cake in rabbits (10), while another phytogenic rabbit study recorded concurrent changes in villus height and crypt depth (7). These comparisons indicated that dietary plant ingredients can influence intestinal architecture, but they do not establish that all such changes have the same biological meaning. Higher villi are often interpreted as a potentially favorable adaptation for nutrient absorption, whereas deeper crypts may reflect increased epithelial proliferation and renewal (35). However, crypt hyperplasia can also occur in response to greater mucosal turnover. Studies in weaned piglets show that nutrition can alter villus height and crypt depth together and that morphometric indices must be interpreted alongside functional and histopathological evidence (36). Because the ratio decreased in *O. ficus-indica* powder rabbits, the current pattern should be described as duodenal remodelling or adaptation, not as an unequivocal improvement in mucosal health. The unchanged growth performance and the absence of reported clinical impairment argue against a major adverse effect, but assessment of inflammatory infiltrates, epithelial integrity, goblet cells, proliferation markers, and tight-junction proteins would be needed to define the biological significance of the deeper crypts. Accordingly, the lower villus height-to-crypt depth ratio could reflect proportionally greater crypt-cell proliferation or epithelial turnover and cannot, by itself, be equated with enhanced absorptive function or barrier integrity.

## 5 Conclusion

Dietary inclusion of dried *O. ficus-indica* cladode powder at 50 g/kg feed was well tolerated by growing rabbits and did not impair growth performance, feed efficiency, slaughter traits, or the proximate composition of meat. The supplementation selectively improved crude fiber and neutral detergent fiber digestibility and was associated with higher circulating antioxidant capacity, lower lipid peroxidation, reduced serum cholesterol and triglycerides, fewer cultivable caecal *E. coli*, and more *Lactobacillus* spp. The duodenal findings indicated mucosal remodelling, with higher villi and deeper crypts and a lower villus height-to-crypt depth ratio; however, these changes should not be interpreted as evidence of improved intestinal health without complementary functional or histopathological markers. Overall, the findings identified 5% dried cladode powder as a promising functional ingredient for growing-rabbit diets, with benefits expressed primarily in fiber utilization and selected metabolic and microbial indicators rather than in productive performance. However, interpretation is based on a single inclusion level; the dose–response relationship, minimum effective dose, and optimal inclusion level cannot be established, and further feeding trials are in progress.

## Data Availability

The raw data supporting the conclusions of this article will be made available by the authors, without undue reservation.
